# Health-related quality of life and healthcare consultations among adult patients before and after diagnosis with rheumatic heart disease in Namibia

**DOI:** 10.1186/s12872-023-03504-4

**Published:** 2023-09-14

**Authors:** Panduleni Penipawa Shimanda, Stefan Söderberg, Scholastika Ndatinda Iipinge, Lars Lindholm, Fenny Fiindje Shidhika, Fredrik Norström

**Affiliations:** 1 Department of Epidemiology and Global Health, Umeå University, 901 87 Umeå, SE Sweden; 2 Clara Barton School of Nursing, Welwitchia Health Training Centre, Pelican Square, P.O. Box 1835, Windhoek, Namibia; 3 Department of Public Health and Clinical Medicine, Umeå University, 901 87 Cardiology, Umeå, SE Sweden; 4 Department of Cardiology, Windhoek Central Hospital, Windhoek, Namibia

**Keywords:** Health-related quality of life, RHD, QALY, EQ-5D-5L, Namibia

## Abstract

**Background:**

Rheumatic Heart Disease (RHD) causes high morbidity and mortality rates among children and young adults, impacting negatively on their health-related quality of life (HRQoL). This study aimed to evaluate the HRQoL and healthcare consultations of adult patients with RHD in Namibia.

**Methods:**

From June 2019 to March 2020, a questionnaire was administered to 83 RHD patients during routine follow-ups. The EQ-5D-5L instrument was used to assess the health-related quality of life before diagnosis and at the time of the survey. The Ethiopian value set for EQ-5D-5L was used to calculate Quality-Adjusted Life Years (QALY).

**Results:**

Most respondents were women (77%), young adults below the age of 30 years (42%), and individuals who grew up in rural areas (87%). The mean QALY statistically significantly improved from 0.773 pre-diagnosis to 0.942 in the last 12 months (*p* < 0.001). Sixty-six patients who had surgery reported a better QALY. Healthcare visits statistically significantly increased from on average 1.6 pre-diagnosis to 2.7 days in the last 12 months (*p* < 0.001). The mean distance to the nearest facility was 55 km, mean cost of transport was N$65, and mean time spent at the clinic was 3.6 h. The median time from diagnosis to the survey was 7 years (quartiles 4 and 14 years).

**Conclusion:**

Treatment and surgery can improve HRQoL substantially among RHD patients. Being diagnosed with RHD affects patients living in socioeconomically disadvantaged rural areas through cost and time for healthcare visits. It would be valuable with further research to understand differences between disease severities.

## Background

Health-related quality of life (HRQoL) is a multidimensional value that reflects an individual's self-perceived health status, modified by impairments, functional status, perceptions, and social opportunities affected by disease, injury, treatment, or policy [1, 2]. HRQoL is crucial in healthcare, assisting clinicians in informed treatment decisions, calculating quality-adjusted life-years (QALY) for economic evaluations and healthcare resource allocation [3].

Rheumatic Heart Disease (RHD) is a condition that can have a significant impact on the patients’ quality of life. RHD is caused by inflammation of heart valves, leading to fibrotic changes and avascularised tissues, resulting in chronic RHD [4–6]. The mitral valve is the most commonly affected, but mixed valvular damage is also common [7, 8].

Chronic RHD can cause various complications, including heart failure, atrial fibrillation, subacute bacterial endocarditis, stroke, poor maternal outcomes, progressive morbidity/disability, reduced quality of life, and premature mortality [9]. In addition, patients face various psychosocial challenges, including pain from Benzathine Penicillin injections, emotional and psychological struggles, stigma, and human relationship issues [10–14]. Therefore, these complications and RHD-related progressive morbidity can adversely impact the individual’s HRQoL [15–22].

The treatment plan for RHD includes chronic medication to manage symptoms, as well as monthly intramuscular Benzathine Penicillin for secondary prophylaxis to prevent the recurrence of ARF [18]. Health care consumption increases due to routine treatment consultations, and patients may incur transportation costs and forego productive time. These socioeconomic challenges can exacerbate compliance issues with treatment and prophylaxis, which are vital for managing disease morbidity [23–25].

RHD remains a neglected global health concern affecting approximately 40.5 million people and is associated with 300,000 deaths annually, predominantly children and women of reproductive age [26, 27]. RHD is most prevalent in socially disadvantaged communities, where social determinants of health such as overcrowding, poor sanitation, and inequitable access to healthcare are contributing factors in the aetiology of ARF and RHD, in addition to genetic predisposition [28–30].

**Fig. 1:**
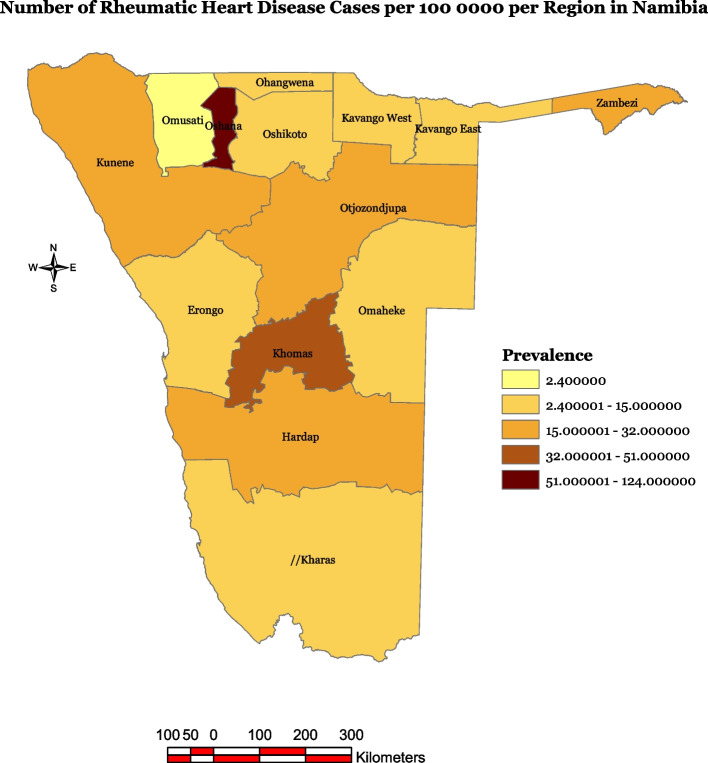
Namibian map and distribution of RHD cases in Namibia [31]

There is limited data available on the prevalence of RHD in Namibia. Overall, estimates suggest it affects about 1% of the population, but recent evidence suggest it may be as low as 0.05–0.1% of the population [31]. RHD is one of the top three causes of cardiovascular death in children ages 5–14, along with congenital heart disease [32]. It is more common among women and children in the northern regions of the country, particularly in socially disadvantaged vast rural areas with limited access to healthcare (Fig. 1) [31, 32]. RHD patients need to travel to the nearest health facility at least once a month for medicine and prophylaxis injections, and they visit the cardiac clinic regularly for assessments with a cardiologist, and psychosocial support.

Our study aims to assess the HRQoL and healthcare consultations among adult RHD patients in Namibia before and after diagnosis.

## Methodology

The study was conducted in Namibia, a sparsely populated country in Southern Africa with 2.6 million inhabitants (Fig. 1). The government's general health expenditure is approximately 8.5% of the gross domestic product, which amounts to US$ 4,179.3 per capita [33].

We collected survey data between June 2019 to March 2020 at the public outpatient cardiac clinic at Windhoek Central Hospital and the satellite outpatient outreach clinic at Intermediate Hospital Oshakati. Windhoek Central Hospital is the only public tertiary hospital that provides specialised cardiac care services, including routine follow-ups such as assessment by cardiologists, health education, and nursing care support.

RHD patients who visited the clinic during the study period were invited to participate. Only patients aged 18 years and older who provided informed consent were enrolled in the study. All patients with RHD had been diagnosed by a cardiologist.

Participants received a self-administered questionnaire, and the researcher was present to explain and interpret questions if necessary. The first section of the questionnaire collected data on sociodemographic and clinical characteristics, such as whether the participant had undergone surgery. The second section asked about the frequency of healthcare visits and admissions, missed working/school days, distance to health facility, mode of transport, and duration of stay at the facility. Participants were required to provide retrospective information for the year before their RHD diagnosis and the last 12 months before the survey.

The EuroQol 5 dimensions instrument with 5 response options (EQ-5D-5L) developed by the EuroQol group was utilized to measure HRQoL [34]. The questionnaire requested responses for the year prior to RHD diagnosis and at the time of the survey. The first part of EQ-5D-5L comprises five dimensions (Mobility, Self-care, Usual activities, Pain or discomfort, and Anxiety or depression), each having five response levels that correspond to no problems, slight, moderate, severe, and extreme problems. The second part of EQ-5D-5L comprises a visual analogue scale (VAS), where patients rate their quality of life on a scale ranging from 0 to 100.

Heart valve diseases were classified into four categories: (i) mitral, (ii) aortic, (iii) tricuspid, or (iv) a combination of these. Mitral valve disease was defined as having mitral regurgitation, mitral stenosis, or a combination of both with tricuspid regurgitation, or stenosis. A similar definition was applied for aortic valve disease. Tricuspid valve disease was defined as exhibiting either tricuspid regurgitation or stenosis alone. Mixed valve disease applied to individuals with both aortic and mitral disease, aortic and tricuspid disease, or mitral and tricuspid disease.

Responses from the EQ-5D-5L questionnaire are presented by dimension for each patient group and for subgroups. The responses are then transformed into a Quality-Adjusted Life Year (QALY) score using the Ethiopian population EQ-5D value set in a decremental approach [35]. The QALY score measures health-related quality of life, anchored at 0, which corresponds to death, and 1, which corresponds to full health. The Ethiopian tariff was considered the most suitable for Namibia as it is from a country in Sub-Saharan Africa.

Survey data was captured and managed using Office 365 Microsoft Access and Excel before exporting to STATA version 14.2 for analysis. Descriptive analyses were presented as percentages, means, medians with standard deviations (SD), and 25^th^ and 75^th^ quartiles. Pairwise comparisons of the QALY scores before diagnosis and at the time of the study were performed using the Wilcoxon signed-rank test. Mann–Whitney *U* rank sum tests were used to compare QALY scores between groups. Costs are presented in Namibian dollars (1 USD = 18.6 NAD, 13^th^ of July 2023).

The study was conducted in accordance with ethical principles outlined in the World Medical Association Helsinki Declaration. Ethical approval was obtained from the Biomedical Research Ethics Committee (BREC) and Research Management Committee (RMC) (FWA No.: FWA00029587) at the Namibian Ministry of Health and Social Services (Study Approval Reference: 17/3/3 PPS). Permission for data collection was then obtained from each hospital's superintendent. Informed consent forms were obtained from all study participants after informing them about the study objectives and assuring them that their participation was voluntary, and there would be no prejudice for refusal or withdrawal. Patients were given the opportunity to ask questions before signing the informed consent form. There were no incentives for participation in the study, nor did participation influence the care provided.

## Results

Eighty-three adult patients with clinical RHD responded to the survey. Table 1 presents the patients’ characteristics. The majority of participants were women (77%), young adults between 20 and 29 years old (42%), grew up in rural areas (87%), completed secondary school education or higher (79%), and were unemployed (51%). Mixed valve disease (35%) and mitral valve disease (34%) were more common than aortic valve disease (17%). The majority of patients (84%) underwent surgery for heart valve repair and/or replacement. The median time from the surgery to the survey was 7 years (interquartile range 3 to 9 years), and the mean time was 7 years (standard deviation 5 years). Similarly, the median time from diagnosis to the survey was 7 years (interquartile range 4 to 14 years), and the mean time was 10 years (standard deviation 8 years).

**Table 1:** Characteristics and Quality-Adjusted Life Year (QALY) of patients with rheumatic heart disease pre-and post-diagnosis

Qaly Prior Diagnosis				Qaly At Time Of The Survey			
**Characteristic**	**n (%)**	**Mean (SD)**	**Median (1st&3rd quartile)**	**n (%)**	**Mean (SD)**	**Median (1st&3rd quartile)**	***p*-value**^**a**^
**All**	78	0.773 (0.317)	0.915 (0.664&0.915)	83	0.941 (0.158)	1 (0.952&1)	< 0.001
**Sex**			***p* = 0.114**^**b**^			***p* = 0.812**^**b**^	
Women	59 (76)	0.741 (0.340)	0.900 (0.576&1)	64 (77)	0.951 (0.112)	1 (0.95&1)	0.002
Men	19 (24)	0.872 (0.212)	1 (0.841&1)	19 (23)	0.906 (0.263)	1 (0.964&1)	0.23
**Age**			**NC**^**1**^			**NC**^**1**^	
18 – 19 years	4 (5)	0.900 (0.159)	0.966 (0.798&1)	4 (4.8)	0.952 (0.748)	0.983 (0.904&1)	NC^2^
20 – 29 years	32 (41)	0.769 (0.321)	0.908 (0.696&1)	35 (42)	0.952 (0.118)	1 (0.964&1)	0.01
30 – 39 years	24 (31)	0.762 (0.257)	0.848 (0.615&1)	25 (30)	0.943 (0.115)	1 (0.964&1)	0.008
40 – 49 years	12 (15)	0.699 (0.482)	0.958 (0.444&1)	13 (16)	0.885 (0.315)	1 (0.904&1)	NC^2^
≥ 50 years	6 (8)	0.904 (0.190)	1 (0.900&1)	6 (7.2)	0.976 (0.029)	0.984 (0.952&1)	NC^2^
**Place of residence**			***p* = 0.918**^**b**^			***p* = 0.813**^**b**^	
Rural	67 (86)	0.782 (0.287)	0.915 (0.664&1)	72 (87)	0.936 (0.169)	1 (0.95&1)	< 0.001
Urban	11 (14)	0.719 (0.476)	0.936 (0.448&1)	11 (13)	0.968 (0.054)	1 (0.968&1)	NC^2^
**Marital status**			***p* = 0.986**^**b**^			***p* = 0.661**^**b**^	
Married/Living together	8 (10)	0.695 (0.419)	0.95 (0.345&1)	8 (10)	0.974 (0.025)	0.971 (0.960&1)	
Single	67 (86)	0.779 (0.310)	0.915 (0.665&1)	71 (90)	0.951 (0.110)	1 (0.964&1)	
**Education**			**NC**^**1**^			**NC**^**1**^	
No formal education	2 (3)	1	1 (1&1)	2 (2.4)	0.984 (0.228)	0.984 (0.968&1)	NC^2^
Primary education	15 (19)	0.851 (0.226)	0.948 (0.772&1)	16 (19)	0.955 (0.082)	1 (0.926&1)	0.17
Secondary education	49 (63)	0.725 (0.345)	0.896 (0.514&1)	52 (63)	0.922 (0.193)	0.987 (0.940&1)	0.003
Tertiary education	12 (15)	0.833 (0.296)	0.949 (0.792&1)	13 (16)	0.991 (0.014)	1 (0.977&1)	NC^2^
**Employment**			**NC**^**1**^			**NC**^**1**^	
Employed/Self employed	31 (40)	0.724 (0.377)	0.948 (0.484&1)	34 (41)	0.954 (0.103)	1 (0.966&1)	0.01
Student	7 (9)	0.749 (0.413)	0.915 (0.665&1)	7 (8.0)	0.951 (0.059)	0.966 (0.915&1)	NC^2^
Unemployed/Retired	40 (51)	0.816 (0.243)	0.908 (0.696&1)	42 (51)	0.928 (0.202)	1 (0.948&1)	0.009
**Smoking**			***p* = 0.728**^**b**^			***p* = 0.469**^**b**^	
Smoker	3 (4)	0.763 (0.215)	0.711 (0.579&1)	3 (4.0)	0.939 (0.080)	0.968 (0.849&1)	
Non-smoker	75 (96)	0.774 (0.322)	0.916 (0.664&1)	80 (96)	0.941 (0.161)	1 (0.958&1)	< 0.001
**Comorbidities**			***p* = 0.132**^**b**^			***p* = 0.186**^**b**^	
Yes	13 (17)	0.916 (0.118)	0.968 (0.841&1)	13 (16)	0.866 (0.316)	0.968 (0.948&1)	NC^2^
No	65 (83)	0.745 (0.337)	0.9 (0.576&1)	70 (84)	0.954 (0.106)	1 (0.964&1)	< 0.001
**Years with RHD**			***p* = 0.645**^**b**^			***p* = 0.158**^**b**^	
< 10 years	50 (64)	0.769 (0.329)	0.934 (0.664&1)	53 (64)	0.917 (0.194)	1 (0.93&1)	0.019
≥ 10 years	28 (36)	0.781 (0.301)	0.908 (0.681&1)	30 (36)	0.982 (0.027)	1 (0.968&1)	< 0.001
**Surgery**			***p* = 0.084**^**b**^			***p* = < 0.007**^**b**^	
Yes	66 (85)	0.747 (0.335)	0.900 (0.576&1)	70 (84)	0.962 (0.093)	1 (0.966&1)	< 0.001
No	12 (15)	0.916 (0.126)	1 (0.827&1)	13 (16)	0.824 (0.322)	0.964 (0.842&0.974)	NC^2^
**Years after Surgery**			***p* = 0.144**^**b**^			***p* = 0.096**^**b**^	
< 10 years	53 (85)	0.792 (0.274)	0.915 (0.665&1)	56 (81)	0.956 (0.103)	1 (0.958&1)	0.001
≥ 10 years	12 (15)	0.658 (0.366)	0.841 (0.368&0.957)	13 (19)	0.994 (0.012)	1 (1&1)	NC^2^
**Heart Valve**			**NC**^**1**^			**NC**^**1**^	
Mitral	31 (40)	0.660 (0.338)	0.727 (0.370&1)	34 (41)	0.942 (0.139)	1 (0.966&1)	< 0.001
Aortic	14 (18)	0.913 (0.180)	1 (0.900&1)	14 (17)	0.938 (0.083)	0.966 (0.934&1)	NC^2^
Mixed	33 (42)	0.820 (0.313)	0.964 (0.772&1)	35 (42)	0.94 (0.198)	1 (0.968&1)	0.032

Two-sample Wilcoxon rank-sum (Mann–Whitney) test

*QALY* Quality Adjusted Life Years

*SD* Standard deviation

*NC*^*1*^* (No comparison)* Comparison for variables with more than two outcomes are not conducted

*NC*^*2*^* (No comparison)* Comparison pre-and post-diagnosis within groups are not conducted if fewer than 15 patients

^a^Comparison of QALY before diagnosis and at today between groups e.g., men and women

^b^Comparison of QALY before diagnosis and at today

Table 2 presents a summary of the EQ-5D-5L responses. The most common response, both pre-diagnosis and at present, across all five dimensions was “no problem.” Approximately 62% of patients reported experiencing at least some problems (levels 2, 3, 4, 5) in at least one dimension before diagnosis, compared to 45% at present. The mobility (*n* = 23), usual activities (*n* = 19), and pain/discomfort (*n* = 16) dimensions showed the greatest improvement between the year before diagnosis and the time of the study.

**Table 2:** EuroQol-5D-5L responses the year prior RHD diagnosis (*n* = 78) and during the survey (*n* = 83)

Dimensions	Level responses the year Prior diagnosis of RHD (*n* = 78)		Level responses at during the survey (*n* = 83)	
	**n**	**%**	**n**	**%**
**Mobility**				
No problems	42	54	73	88
Slight problems	10	13	7	8.4
Moderate problems	15	19	2	2.4
Severe problems	8	10	0	
Extreme problems	3	4	1	1.2
**Self-care**				
No problems	53	68	78	94
Slight problems	8	10	2	2.4
Moderate problems	8	10	2	2.4
Severe problems	7	9	0	
Extreme problems	2	3	1	1.2
**Usual activities**				
No problems	41	53	60	72
Slight problems	9	12	14	17
Moderate problems	9	12	6	7.2
Severe problems	11	14	2	2.4
Extreme problems	8	10	1	1.2
**Pain/Discomfort**				
No problems	40	51	68	82
Slight problems	13	17	6	7.2
Moderate problems	12	15	7	8.4
Severe problems	10	13	2	2.4
Extreme problems	3	4	0	
**Anxiety/Depression**				
No problems	62	79	71	86
Slight problems	7	9	6	7.2
Moderate problems	6	8	4	4.8
Severe problems	1	1	0	
Extreme problems	2	3	2	2.4

There was a statistically significant improvement in QALY from RHD diagnosis (mean QALY of 0.773) to the time of response (mean QALY of 0.941) (*p* < 0.001, quartiles 0.113 and 0.301). Patients who underwent surgery had a significantly improvement in the QALY (0.747) prior diagnosis compared to the QALY (0.962) (*p* < 0.001) at the time of survey. The mean QALY decreased among patients who did not undergo surgery from 0.946 prior diagnosis to 0.824 at the time of survey.

The EQ-VAS rating (Table 3) statistically significantly improved from 66 at the time of diagnosis to 79 at the time of the study (*p* = 0.005). Moreover, the EQ-VAS rating demonstrated a significant improvement among patients who underwent surgery prior diagnosis compared to rating at the time of the survey (*p* = 0.005).

**Table 3:** Characteristics and EQ visual analogue scale (VAS) number for patients with rheumatic heart disease pre-and post-diagnosis

VAS Prior Diagnosis				VAS At Time Of The Survey			
**Characteristic**	**n (%)**	**Mean (SD)**	**Median (1st&3rd quartile)**	**n (%)**	**Mean (SD)**	**Median (1st&3rd quartile)**	***p*-value**^**a**^
**All**	78	66 (30)	73 (40&95)	81	79 (17)	80 (70&95)	0.005
**Sex**			***p* = 0.121**^**b**^			***p* = 0.882**^**b**^	
Women	60 (77)	63 (31)	60 (35&95)	63 (78)	80 (17)	80 (70&95)	0.002
Men	18 (23)	77 (26)	85 (55&100)	18 (22)	78 (21)	80 (55&99)	0.983
**Age**			**NC**^**1**^			**NC**^**1**^	
18 – 19 years	4 (5)	79 (10)	78 (70&88)	4 (5)	88 (19)	95 (75&100)	NC^2^
20 – 29 years	31 (40)	63 (30)	55 (30&95)	33 (41)	80 (17)	80 (70&95)	0.004
30 – 39 years	25 (32)	62 (29)	50 (35&95)	25 (31)	84 (13)	85 (80&95)	0.009
40 – 49 years	12 (15)	63 (74)	74 (39&93)	13 (16)	72 (22)	70 (50&90)	NC^2^
≥ 50 years	6 (8)	100 (0)	100 (100&100)	6 (7)	63 (12)	65 (50&70)	NC^2^
**Place of residence**			***p* = 0.971**^**b**^			***p* = 0.846**^**b**^	
Rural	67 (86)	66 (31)	75 (35&100)	70 (86)	79 (18)	80 (70&95)	0.182
Urban	11 (14)	67 (26)	60 (45&95)	11 (14)	80 (17)	80 (75&95)	NC^2^
**Marital status**			***p* = 0.226**^**b**^			***p* = 0.229**^**b**^	
Married/Living together	8 (10)	76 (32)	95 (78&100)	8 (10)	74 (15)	75 (65&80)	NC
Single	67 (86)	65 (30)	70 (35&95)	69 (85)	80 (17)	80 (70&95)	0.002
**Education**			**NC**^**1**^			**NC**^**1**^	
No formal education	2 (3)	78 (32)	78 (55&100)	2 (2)	100 ()	100 (100&100)	NC^2^
Primary education	14 (18)	83 (24)	95 (75&100)	15 (19)	78 (20)	80 (50&100)	NC^2^
Secondary education	49 (63)	62 (31)	60 (35&90)	51 (63)	78 (17)	80 (60&95)	0.006
Tertiary education	13 (17)	64 (29)	55 (45&90)	13 (16)	82 (14)	85 (70&90)	NC^2^
**Employment**			**NC**^**1**^			**NC**^**1**^	
Employed/Self employed	29 (38)	67 (30)	75 (45&95)	31 (38)	81 (17)	80 (70&99)	0.076
Student	7 (10)	61 (27)	70 (30&90)	7 (9)	81 (15)	85 (60&95)	NC^2^
Unemployed/Retired	42 (54)	66 (31)	75 (35&100)	43 (53)	78 (18)	80 (60&95)	0.047
**Smoking**			***p* = 0.647**^**b**^			***p* = 0.659**^**b**^	
Smoker	3 (4)	75 (31)	85 (40&100)	3 (7)	74 (26)	80 (45&96)	NC^2^
Non-smoker	75 (96)	66 (30)	70 (40&95)	78 (96)	79 (17)	80 (70&95)	0.004
**Comorbidities**			***p* = 0.368**^**b**^			***p* = 0.625**^**b**^	
Yes	12 (15)	72 (32)	85 (40&100)	12 (15)	76 (20)	80 (55&93)	NC^2^
No	66 (85)	65 (30)	70 (40&95)	69 (85)	80 (17)	80 (70&95)	0.003
**Years with RHD**			***p* = 0.323**^**b**^			***p* = 0.081**^**b**^	
< 10 years	50 (64)	69 (30)	78 (40&100)	52 (64)	77 (18)	80 (60&90)	0.154
≥ 10 years	28 (36)	62 (31)	55 (30&93)	29 (34)	84 (16)	90 (70&99)	0.005
**Surgery**			***p* = 0.925**^**b**^			***p* = 0.005**^**b**^	
Yes	67 (86)	66 (30)	75 (40&95)	69 (85)	81 (17)	85 (70&95)	0.003
No	11 (14)	67 (31)	70 (35&100)	12 (15)	66 (14)	65 (53&78)	NC^2^
**Years after Surgery**			***p* = 0.731**^**b**^			***p* = 0.216**^**b**^	
< 10 years	54 (82)	67 (30)	80 (40&95)	55 (81)	81 (17)	80 (70&95)	0.021
≥ 10 years	12 (18)	60 (31)	50 (45&98)	13 (19)	86 (14)	90 (80&100)	NC^2^
**Heart Valve**			**NC**^**1**^			**NC**^**1**^	
Mitral	31 (40)	58 (31)	49 (30&90)	34 (42)	80 (17)	80 (70&95)	0.002
Aortic	14 (18)	85 (28)	98 (86&100)	14 (17)	82 (16)	85 (70&99)	NC^2^
Mixed	33 (42)	66 (27)	67 (40&90)	33 (41)	77 (19)	80 (60&95)	0.131

Two-sample Wilcoxon rank-sum (Mann–Whitney) test

*VAS* EQ Visual Analogue Scale

*SD* Standard deviation

*NC*^*1*^* (No comparison)* Comparison for variables with more than two outcomes are not conducted

*NC*^*2*^* (No comparison)* Comparison pre-and post-diagnosis within groups are not conducted if fewer than 15 patients

^a^Comparison of EQ VAS before diagnosis and at today between groups e.g., men and women

^**b**^Comparison of EQ VAS before diagnosis and at today

Overall, there was insufficient evidence to conclude statistically significant changes in QALYs based on sex, place of residence, and comorbidities. However, statistically significant changes were observed among women (mean QALY increasing from 0.741 to 0.951) (*p* = 0.002), individuals residing in rural areas (mean QALY increasing from 0.782 to 0.936) (*p* = 0.001), and those without comorbidities (mean QALY increasing from 0.745 to 0.954) (*p* < 0.001).

**Fig. 2:**
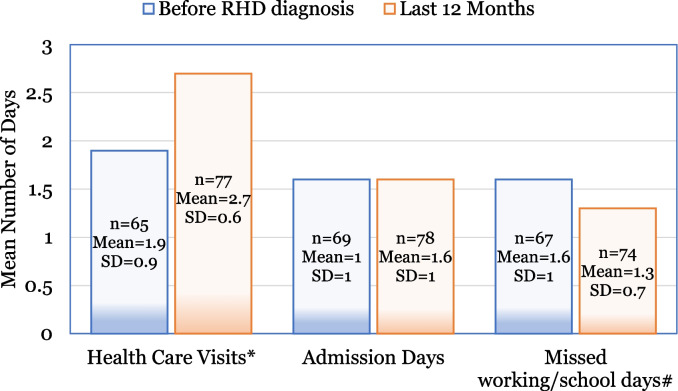
Healthcare consultations among the RHD patients. * *p* < 0.001, # *p* = 0.138, SD = Standard Deviation

There was a statistically significant increase in the number of visits to healthcare facilities (Fig. 2), from 1.6 days prior to diagnosis to 2.7 days in the last 12 months (*p* < 0.001). On average, patients missed 1.6 working or school days before diagnosis, which decreased to 1.3 days in the last 12 months, though this change was not statistically significant (*p* = 0.138).

The distance to the nearest healthcare facility ranged from 1 to 250 km (Table 4). Sixty-one percent of the patients had to travel at least 20 km to reach the facility, with a median distance of 10 km (interquartile range 5 to 30 km) and a mean distance of 55 km (standard deviation 189 km). The majority of these patients (78%) travelled by paid transport and spent at least N$24, with a median travel time of 2 h (interquartile range 1 to 3 h) and a mean travel time of 2.1 h (standard deviation 1.2 h). Similarly, the median cost was N$34 (first quartile N$24 and third quartile N$75), and the mean cost was N$65 (standard deviation N$99).

**Table 4:** Factors regarding patient consultations for RHD care

Characteristic	n (%)	Mean (SD)	Median (1st&3rd quartile)
**Distance to health facility (*n* = 67)**		**55 km (189)**	**10 km (5&30)**
0–9 km	24 (29)		
10–19 km	18 (22)		
20–29 km	7 (8.0)		
≥ 30 km	34 (41)		
**Travel time to health facility (*n* = 82)**		**2.1 h (1.2)**	**2 h (1&3)**
< 30 min	34 (41)		
30–60 min	19 (23)		
1–2 h	16 (20)		
2–3 h	10 (12)		
> 3 h	3 (4.0)		
**Transport to health facility (*n* = 83)**			
Own car	7 (8.4)		
Taxi	65 (78)		
Bicycle	1 (1.2)		
Walking	10 (12)		
**Cost of transport to health facility (*n* = 83)**		**N$ 65 (99)**	**N$ 34 (24&75)**
No cost	18 (22)		
≤ N$ 24	30(36)		
N$ 25–29	7 (8)		
≥ N$ 50	28 (34)		
**Time spent at health facility (*n* = 78)**		**3.6 h (2)**	**4 h (2&5)**
1 h	15 (19)		
2–3 h	19 (24)		
4–5 h	32 (41)		
> 5 h	12 (15)		

*km* Kilometres

*N$* Namibian Dollar

Fifty-six percent of the patients reported spending four or more hours at the healthcare facility for their RHD care, with a median time of 4 h (interquartile range 2 to 5 h) and a mean time of 3.6 h (standard deviation 2 h).

## Discussion

In our study, we found that Namibian RHD patients experienced a substantial improvement in their quality of life after receiving treatment, particularly among those who had undergone surgery. This improvement was likely due to the clinical recovery from surgery and secondary prophylaxis, which can improve the clinical condition of RHD to an asymptomatic state, as shown in previous studies [15, 36, 37]. Our study results showed a good QALY after initiated treatment despite the challenges that RHD patients face, such as pain from monthly prophylaxis injections and psychosocial and economic limitations [10–14]. From our study, it is difficult to draw conclusions based on surgery, as only 13 participants had not undergone surgery. Our study suggests that without surgery health might gradually deteriorate, likely due to valvular disease progression [38].

The low reported QALY during the year before RHD diagnosis may be due to living with undiagnosed subclinical or symptomatic RHD. This is likely due to the persistent challenges in detecting and diagnosing RHD, especially in low-middle income settings with limited cardiac expertise and diagnostic resources [4, 32].

Compared to similar studies using the EQ-5D instrument, our study showed a high QALY score. In South Africa, a QALY of 0.848 was reported among 48 adult RHD patients without surgery [39], while in India, a QALY score of 0.820 was reported among adult RHD patients [17]. Our study adds to the literature by comparing pre-diagnosis and post-treatment situations, which may explain the improved QALY scores. Similar to Dixit et al.’s [17] findings, there were no observable differences in sociodemographic characteristics, but differences were noted among women and those living in rural areas.

Our study consisted mostly of young adult women from rural areas in northern Namibia, reflecting current knowledge about RHD prevalence [5, 29, 40–42]. We found that rural residents face additional healthcare costs related to transportation, highlighting the socioeconomic impact of RHD on patients in poor settings and the inequities in healthcare access [32, 43]. To reduce these inequities, decentralisation of RHD services, along with outreach visits and healthcare worker training, could be implemented. Community education efforts are also crucial to ensure effective diagnosis and management.

A strength of our study is that it is one of the few that compares QALYs in assessing HRQoL among adult RHD patients, which is valuable for cost-effectiveness studies [17, 39]. Responses to HRQoL before diagnosis might be affected by recall bias and patients might be overreporting their problems. Patients responded to the questionnaire before they received follow-up care for their RHD. It could be argued that this would positively affect their responses as they were about to get support for their disease. The follow-up care is usually determined well in advance, still, it might be so that they are more likely to visit healthcare facility for follow-up due to their health. Thus, a bias could be in either direction. Some patients might be poor in following up their RHD. The above-mentioned issues could bias our results. However, we are confident that such bias would at most be modest. Considering the large improvement after diagnosis and treatment such bias should not affect our conclusions. The small sample size is a limitation and larger studies would be beneficial to conduct in the future. However, we still consider the study large enough to support our main conclusions.

## Conclusion

This study provides valuable insights into the HRQoL experienced by RHD patients before diagnosis and suggests that pharmacological treatment and surgery can improve their quality of life. Additionally, the findings highlight the impact of RHD on patients living in socioeconomically disadvantaged rural areas through cost and time for healthcare visits. The findings underscore the importance of addressing this condition to improve the lives of those affected. It would be valuable with further research to understand differences between disease severities.

## Data Availability

Survey data and materials are available from the corresponding author upon a reasonable request.

## Abbreviations

ARF: Acute Rheumatic Fever
A.S.A.P.: Awareness Surveillance Advocacy Prevention
GAS: Group A Streptococcus
HRQoL: Health-Related Quality of Life
QALY: Quality Adjusted Life Year
RF: Rheumatic Fever
RHD: Rheumatic Heart Disease
VAS: Visual Analogue Scale
